# Experimental demonstration of photon upconversion via cooperative energy pooling

**DOI:** 10.1038/ncomms14808

**Published:** 2017-03-15

**Authors:** Daniel H. Weingarten, Michael D. LaCount, Jao van de Lagemaat, Garry Rumbles, Mark T. Lusk, Sean E. Shaheen

**Affiliations:** 1 Department of Physics, University of Colorado Boulder, Boulder, Colorado 80309, USA; 2 Department of Physics, Colorado School of Mines, Golden, Colorado 80401, USA; 3 Chemistry and Nanoscience Center, National Renewable Energy Laboratory, Golden, Colorado 80401, USA; 4 Department of Chemistry and Biochemistry, University of Colorado Boulder, Boulder, Colorado 80309, USA; 5 Chemistry and Nanoscience Center, Renewable and Sustainable Energy Institute, University of Colorado Boulder, Boulder, Colorado 80309, USA; 6 Department of Electrical, Computer, and Energy Engineering, University of Colorado Boulder, Boulder, Colorado 80309, USA

## Abstract

Photon upconversion is a fundamental interaction of light and matter that has applications in fields ranging from bioimaging to microfabrication. However, all photon upconversion methods demonstrated thus far involve challenging aspects, including requirements of high excitation intensities, degradation in ambient air, requirements of exotic materials or phases, or involvement of inherent energy loss processes. Here we experimentally demonstrate a mechanism of photon upconversion in a thin film, binary mixture of organic chromophores that provides a pathway to overcoming the aforementioned disadvantages. This singlet-based process, called Cooperative Energy Pooling (CEP), utilizes a sensitizer-acceptor design in which multiple photoexcited sensitizers resonantly and simultaneously transfer their energies to a higher-energy state on a single acceptor. Data from this proof-of-concept implementation is fit by a proposed model of the CEP process. Design guidelines are presented to facilitate further research and development of more optimized CEP systems.

## Abstract (web summary)

Photon upconversion methods demonstrated thus far involve challenging requirements. Here Weingarten *et al*. demonstrate a mechanism called cooperative energy pooling, in which multiple photoexcited sensitizers resonantly and simultaneously transfer their energies to a higher-energy state on a single acceptor.

Photon upconversion, the process of combining multiple low-energy photons into one higher-energy photon, has been modelled and studied in various forms since the 1960s (refs 1, 2). The known mechanisms for photon upconversion—namely excited-state absorption, two-photon absorption (2PA), photon avalanche, energy-transfer upconversion (ETU), second harmonic generation, and triplet–triplet annihilation (TTA)—have been observed in atomic, crystalline, nanoparticle and molecular systems^3^, and recent work has demonstrated upconversion yields of up to 38%^4^. Such developments in upconversion have made the technique appealing for a wide range of applications such as deep-tissue photodynamic cancer therapies^5^,^6^, quantum cryptography^7^,^8^, three-dimensional data storage^9^,^10^ and enhanced solar cell devices^11^,^12^. However, there are challenges that face the further development and application of these photon upconversion techniques, including requirements of high-intensity excitation sources, deterioration in ambient conditions, requisite use of exotic elements or phases, or fundamental energy loss steps during the upconversion process.

Cooperative energy pooling (CEP) is an energy transfer mechanism that provides an alternative route towards efficient and applicable photon upconversion. CEP is the process of two photoexcited sensitizer chromophores non-radiatively transferring their energy to a single higher-energy state in an acceptor chromophore. Instead of dipole–dipole coupling between the emissive states of the sensitizers and the absorbing state of the acceptor, as in the Förster resonance energy transfer process (FRET), CEP is carried out via a coupling of the emissive states of both sensitizers with the two-photon absorption (2PA) tensor of the acceptor^13^,^14^,^15^. In this way the sensitizers act as photon storage centres that relax the stringent temporal and spatial constraints for achieving 2PA in the acceptor, enabling upconversion with greater efficiency and at reduced excitation intensities.

There are a number of advantages of CEP over other forms of upconversion. CEP avoids inter-system crossing by utilizing only lowest-lying singlet excitations, allowing for less energy loss and potentially higher-energy yield per upconversion event than processes like TTA. The exclusive use of singlet states also makes CEP unsusceptible to quenching by oxygen and hence makes it more tolerant of environmental conditions than triplet-based mechanisms. While 2PA varies quadratically with excitation intensity and thus becomes negligible in low light conditions, CEP can achieve sub-quadratic intensity dependence by utilizing linearly absorbing sensitizers as antennae to funnel excitations to the acceptor. All upconverted CEP emission occurs from the lowest-lying excited state of the acceptor, thus allowing the use of easily produced organic chromophores as acceptors and avoiding the need for lanthanide nanoparticles typically required in ETU for their ability to emit from highly excited states. The use of organic chromophores also allows for strong absorption over a wide energy range, low environmental and biological toxicity, and the ability to relatively easily design and synthesize sensitizers and acceptors with optimized, arbitrarily tunable properties.

Theoretical work modelling three-body FRET processes^13^,^14^ in the late 1990s laid the foundation for a quantum electrodynamical understanding of the CEP process and more recent computational work^15^ has highlighted the strong dependence of the CEP process on both the separation distance and relative orientations of sensitizer and acceptor chromophores. Experimental work in the 1990s by Nickoleit *et al*.^16^ demonstrated CEP-like energy-upconversion photoisomerization, but the authors were unable to verify the exact mechanism and concluded that it was likely associated with triplet–triplet annihilation.

In this work we demonstrate experimental observation of CEP photon upconversion. We measure the spectroscopic properties of pristine and blended sensitizer and acceptor chromophores and observe significant upconversion in the blended thin film. The mechanism of CEP is verified via measurements of control films with alternative chromophore blends. Further data on the intensity dependence of the observed upconversion process confirms the transition from quadratic towards linear dependence on the excitation intensity, as expected for a multi-chromophore upconversion process. We develop a kinetic model of the CEP process and find good fits to the measured data. This model is then used to present estimates of CEP performance under various conditions, showing that upconversion under solar excitation may be achievable with optimized chromophores. The results of this work are finally brought together to present guidelines for the selection of ideal chromophores for CEP and the development of improved CEP systems.

## Results

### Overview of CEP process

**Figure 1:**
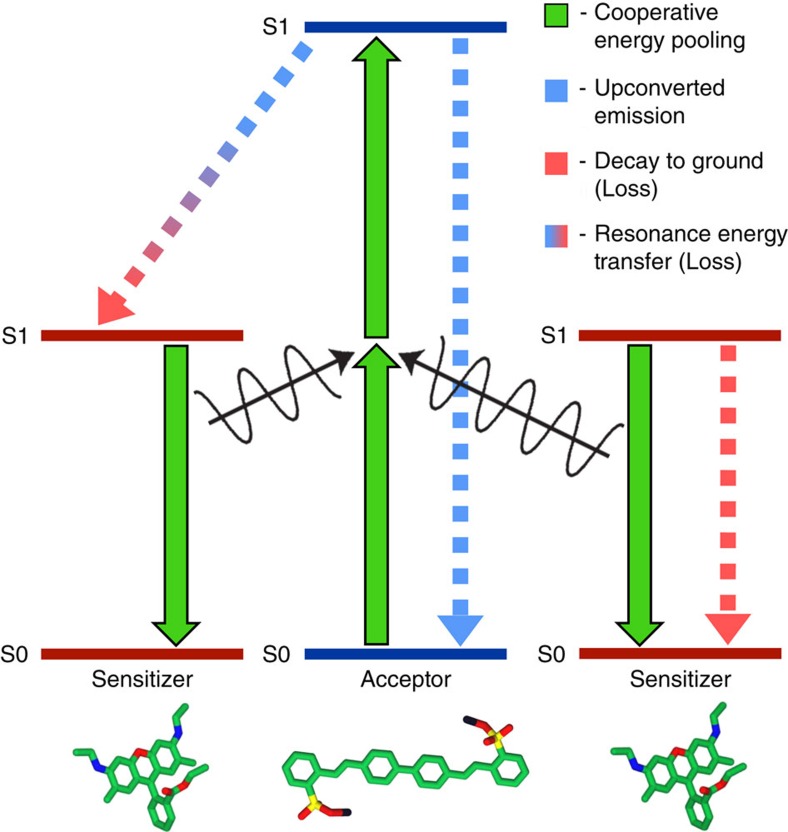
Kinetic diagram of cooperative energy pooling. Two excited sensitizer chromophores (sensitizers) simultaneously transfer their energy to an acceptor chromophore via resonant coupling with the 2PA tensor of the acceptor, resulting in a lowest-lying singlet excitation on the acceptor. S0 and S1 are the ground and first excited singlet states, respectively. Emission of an upconverted photon from the acceptor is measured to detect CEP. Energy loss pathways are shown as FRET from acceptor to sensitizer (blue-to-red arrow) and sensitizer decay (red arrow). Non-radiative decay loss pathways are not shown.

The kinetics diagram of CEP is shown schematically in Fig. 1. The sensitizers absorb low-energy light and can transfer that energy to the acceptor via CEP. The acceptor absorbs high-energy light directly, resulting in a system capable of efficiently harvesting multiple wavelengths of light. Upconversion is observed via radiative emission from the acceptor under low-energy excitation. Though this study demonstrates upconversion in the visible spectrum, the CEP mechanism is achievable with any chromophores that match sensitizer emission with acceptor 2PA energies, allowing this process to be tuned to any desired photon energy range in which suitable chromophores are available. If there is spectral overlap of sensitizer absorption with sensitizer emission bands (that is, small Stokes shift) then FRET and Dexter energy transfer between sensitizers is enabled and exciton migration may occur in the film. Conversely, if there is overlap between the acceptor emission and sensitizer absorption spectra, as there is in the chromophore blend measured in this work, then FRET between acceptors and sensitizers is enabled and functions as a loss pathway by downconverting acceptor excitons into lower energy sensitizer excitons. Other energy loss pathways include non-radiative decay of the acceptor and both radiative and non-radiative decays of the sensitizer.

### CEP upconversion with Stilbene-420 and Rhodamine 6G

To experimentally demonstrate CEP we utilized well-known, commercially available organic chromophores. Stilbene-420 (Stilb420) was chosen as the acceptor due to its sizeable 2PA cross-section^17^ in the 500–600 nm range (Supplementary Fig. 1) while Rhodamine 6G (Rhod6G) was selected as the sensitizer due to its high linear absorptivity, quantum yield near unity^18^ and emission spectrum overlap with the 2PA spectrum of Stilb420.

**Figure 2:**
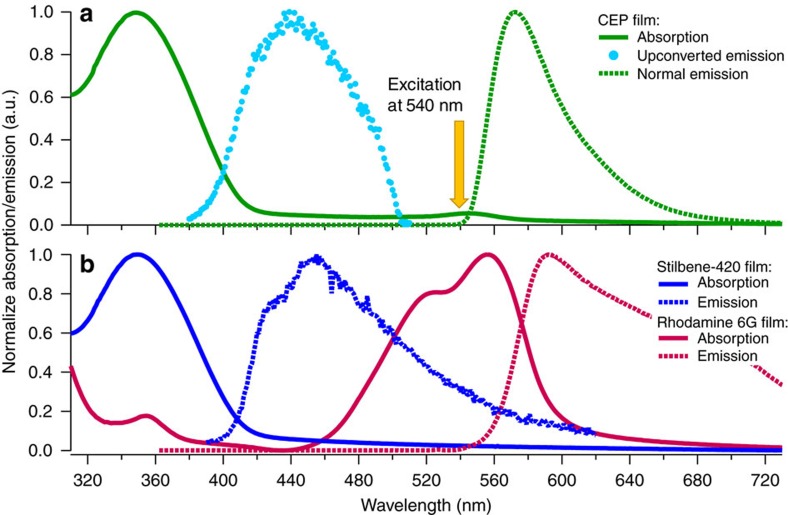
Spectral properties of the materials in this work. (**a**) Absorption, emission and upconverted emission spectra of Rhod6G/Stilb420 blend CEP film. Upconverted emission was measured under 540 nm excitation. Normal emission was measured under excitation at 349 nm to avoid artifacts due to scattered excitation signal overlapping with emission spectrum. (**b**) Absorption and emission spectra of pristine Stilbene-420 (acceptor) and Rhodamine 6G (sensitizer) chromophores in thin films. Emission spectra were measured under excitation at 363 and 525 nm for Stilbene-420 and Rhodamine 6G, respectively.

To fabricate the CEP film, Rhod6G and Stilb420 were combined into solution and blade coated onto glass substrates to yield ∼80 nm thick films. The resulting films had the spectral properties of a linear addition of the two components. Extensive overlap between Stilb420 emission and Rhod6G absorption indicates that any upconversion yields in this system will be strongly reduced by FRET losses from acceptor to sensitizer (Supplementary Fig. 2). Nonetheless, upon excitation with 545 nm light both a normal emission spectrum peaking at 572 nm, corresponding to Rhod6G, and an upconverted emission spectrum peaking at 445 nm, corresponding to Stilb420, were observed (Fig. 2a). Trials with varying sensitizer/acceptor ratios to optimize upconversion yields revealed that Rhod6G exhibits strong self-quenching, necessitating an acceptor-heavy blend and further reducing upconversion yields. See Supplementary Note 1 and Supplementary Fig. 3 for a discussion of chromophore blend ratios and calculations of average sensitizer-acceptor separation distance.

The first evidence for CEP as the mechanism responsible for the observed upconversion is the similarity of the upconverted emission spectrum to the emission of pristine Stilb420. Excitation at photon energies corresponding to sensitizer absorption yielded fluorescence corresponding to acceptor emission, suggesting a multi-body process in which both chromophores play a role in upconversion. The alternative multi-body upconversion processes of ETU and photon avalanche processes may be immediately ruled out since the Stilb420 acceptor has no stable excited states at energies lower than the observed upconverted emission, a requirement of both of those processes.

### Control films to verify CEP mechanism

**Figure 3:**
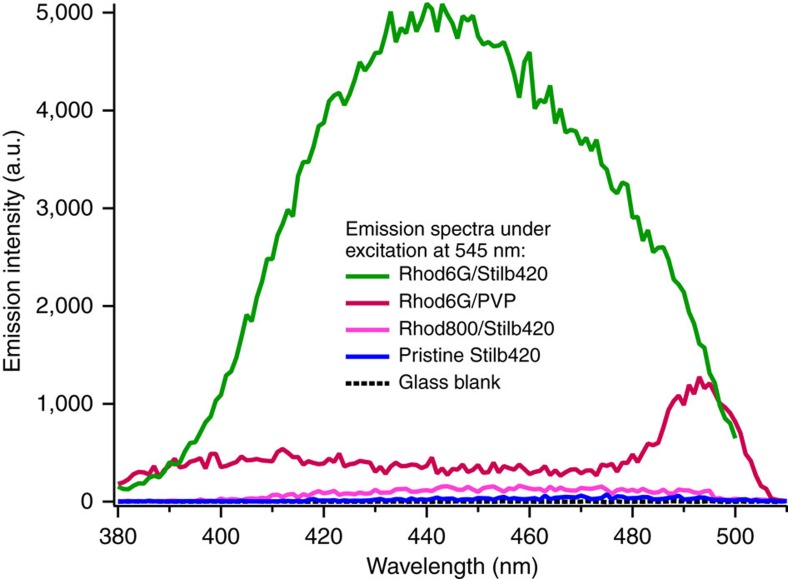
Upconverted emission spectra of CEP film and control films. Upconverted emission spectra of Rhod6G/Stilb420 blend film compared with control films of pristine Stilb420, Rhodamine 800/Stilb420, Rhod6G/PVP and blank glass substrate. Emission from the Rhod6G/PVP film is due to PVP disaggregating Rhod6G and increasing the normal 590 nm emission enough to leak through both short-pass filter and monochromator filtering.

To further verify the upconversion mechanism, control films were made by substituting alternative sensitizers or acceptors in the film. A blend film of neutral host polymer polyvinylpyrrolidone (PVP) and Rhod6G exhibited very strong fluorescence at 570 nm due to disaggregation of the Rhod6G (ref. 19), but no sign of upconversion (Fig. 3). This is expected due to both the negligible two-photon absorption of Rhod6G at the excitation wavelength^20^ and the (sub-picosecond) thermalization of highly excited states down to the lowest-lying excited state in organic chromophores (see Kasha's rule^21^). Additionally, the excited-state absorption spectrum of Rhod6G lies between 400 and 470 nm^22^, indicating that Rhod6G cannot achieve a highly excited state via sequential excitation at the 540 nm excitation wavelength used in this work. Thus, two-photon excitation—either simultaneous or sequential—of the Rhod6G sensitizer and subsequent energy transfer to the Stilb420 acceptor can be eliminated as a potential cause of the observed upconverted emission. Blend films of Stilb420 with Rhodamine 800 (Rhod800), a NIR-emitting analogue of Rhod6G with no emission overlap with the 2PA spectrum of Stilb420, were compared against pristine Stilb420 films to test for doping-induced upconversion improvements. The modest fourfold improvements in Rhod800/Stilb420 films (Fig. 3) indicates that the presence of a molecular dopant does moderately improve 2PA upconversion yields in Stilb420, likely by disaggregating the Stilb420 and hence reducing self-quenching losses. These control films demonstrate that the 160-fold upconversion enhancement in Rhod6G/Stilb420 films must be due to a multi-body, sensitizer-acceptor process and dependent on the spectral overlap between sensitizer emission and 2PA spectrum of the acceptor. Additionally, decay lifetimes for both upconverted and normal fluorescence from the CEP film are distinctly shorter than for the pristine films (Supplementary Fig. 4), indicating strong interaction between sensitizers and acceptors and providing further evidence for energy transfer processes between chromophores—both CEP upconversion and FRET losses. Evidence for a multi-body upconversion process eliminates the possibility of second harmonic generation, excited-state absorption, or 2PA being responsible for the observed upconversion.

Strong evidence supports the conclusion that upconversion in Rhod6G/Stilb420 films is due solely to singlet excited states and is not due to TTA. Rhod6G has an inherently low triplet yield of Φ_T_=0.005 (ref. 23) and all film preparation and measurement was carried out in air, thus ensuring that the low yield of triplets was quenched even further by the presence of atmospheric oxygen. Rhod6G has also been shown to increase triplet yield up to Φ_T_∼0.3 when aggregated^24^, but decreased upconversion was observed at higher loadings of Rhod6G, indicating that an increase in sensitizer triplet population was not beneficial for improving upconversion as would be expected for a TTA system. Finally, lifetime data shows all fluorescence occurring on timescales <1 ns, suggesting that long-lived triplet states are not serving as intermediaries in the upconversion pathway.

Experiments with alternative sensitizer chromophores demonstrate that CEP is a robust process that is reproducible in a variety of chromophore systems. Rhodamine B and Merocyanine 540 have peak emission at 564 and 579 nm, respectively, and were selected as alternatives to Rhodamine 6G due to their reduced overlap with the 2PA spectrum of Stilb420. CEP models predict that reduced spectral overlap between sensitizer and acceptor chromophores should result in reduced upconversion, and this reduction was experimentally verified in blend films even after optimizing for different blend ratios and self-quenching properties. RhodB triplets at 2.05 eV have better overlap with the 2PA spectrum of Stilb420 than the 1.79 eV triplets of Rhod6G (ref. 25), yet Rhod6G/Stilb420 films exhibited better upconversion, further supporting the case for singlet-based CEP being responsible for the observed upconversion.

### Intensity dependence measurements

**Figure 4:**
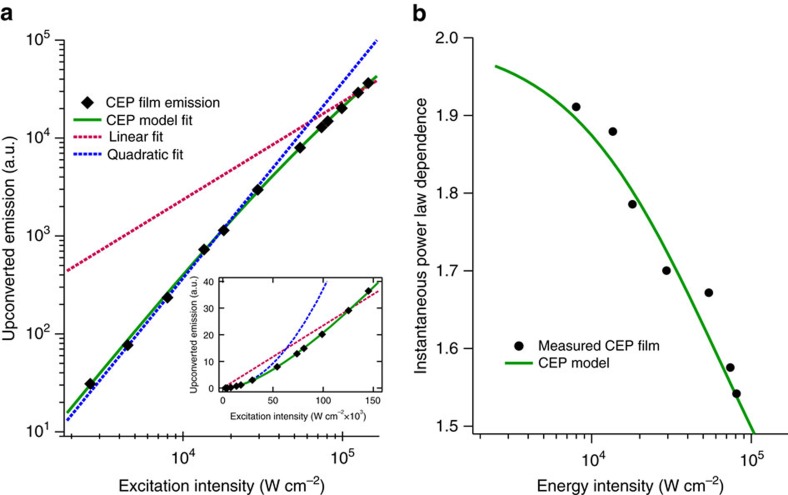
Excitation intensity dependence of the CEP film. (**a**) Log-log plot of upconverted emission from the CEP film at 440±20 nm as a function of 540 nm excitation intensity. The coloured lines are quadratic (blue) and linear (red) fits to the first and last three data points, respectively. The green line is a fit to the CEP kinetic model, discussed below. Excitation-induced film degradation precluded the collection of data at higher excitation intensities. The inset shows the same figure but scaled linearly. (**b**) Instantaneous power-law dependence of measured and modelled excitation dependence curves, showing a progression toward linear power-law dependence at higher excitation intensities. The power-law dependence was determined by the slope of a linear fit to a sliding boxcar window of six data points from the log–log plot of intensity dependence in **a**.

Since CEP is a three-body cooperative energy transfer mechanism involving the addition of two excitons, we expect it to exhibit a similar evolution from quadratic to linear intensity dependence with increasing excitation intensity as observed in TTA literature^26^,^27^,^28^. Measurements of excitation intensity dependence of the CEP film (Fig. 4a) reveal a progression from the quadratic regime into an intermediate regime, as expected. Film degradation at higher excitation intensities prevented measurement into the fully linear regime. Taking the double-logarithm of the intensity dependence data and performing a linear fit to consecutive subsets of neighbouring data points allows for extraction of the instantaneous power-law dependence of CEP upconversion versus excitation intensity (Fig. 4b). This analysis reveals a smooth transition from the quadratic toward the linear regime, analogous to what has been reported in TTA. This transition can be accurately replicated by a kinetic model of the complete CEP process, which is discussed below.

### Modelling the CEP process

To more fully understand the intensity dependence of CEP and explore its feasibility at low excitation intensities we modelled the kinetics of a CEP system, taking into account each of the various possible excitonic pathways. Numerical solutions to the system of differential equations below allowed for simulations of CEP systems with arbitrarily adjustable parameters, enabling us to optimize system parameters *in silico*.


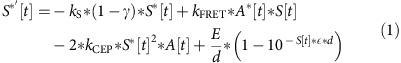



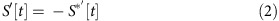



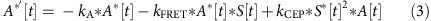



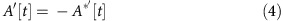



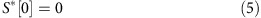



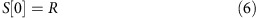



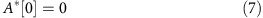



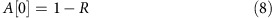


*S*[*t*] and *S*[*t*] are the population densities of the sensitizer in excited and ground states as a function of time *t*, respectively, while *A*[*t*] and *A*[*t*] are the equivalents for acceptor populations. *k*_S_ and *k*_A_ are the rates of sensitizer and acceptor excited-state decay, respectively. *γ* is the likelihood of sensitizer self-reabsorption (for example, homo-FRET). *k*_FRET_ is the rate of FRET from acceptor to sensitizer and *k*_CEP_ is the rate of the CEP process. *E* is the excitation flux intensity, *d* is the thickness of the blend film being modelled, *ɛ* is the molar attenuation coefficient of the sensitizer at the excitation wavelength and *R* is the percentage of the film blend comprised of sensitizer chromophores. Ground state saturation effects are accounted for in this model by including terms for both excited and ground state populations.

Experimental measurements of chromophore lifetime, film thickness, excitation flux, and sensitizer absorbance enable simulated excitation intensity dependence from this CEP model to be fitted quite closely to the experimentally measured data (Fig. 4), yielding estimates for the CEP and FRET rates within the film. Calculating the steady-state population of excited acceptors then allows for an estimate of the quantum yield of the CEP process, both as a function of absorbed photons (internal quantum yield) and incident photons (external quantum yield). Further exploration of the parameters in this model verify that CEP may be achievable at low (near solar AM 1.5) flux intensities. Table 1 lists a number of notable parameter configurations.

## Discussion

From the behaviour of this model with respect to its various parameters, a number of conclusions can be drawn. Firstly, the optimal values for both chromophore blend ratio and film thickness vary as other system parameters are altered. As excitation intensity decreases or sensitizer absorbance increases, the optimal blend ratio skews toward increasingly acceptor-heavy blends and the optimal film thickness decreases. This trend indicates that optimal blend ratio and film thickness parameters are determined by the need for sufficient sensitizers to absorb the excitation light and that any excess sensitizers degrade performance by diluting the concentration of excited sensitizers. Thus, while one might expect a 2:1 blend ratio to be ideal, CEP model simulations suggest that this is only true at high excitation intensities and that blends of 1:1 or lower may be optimal for more typical excitation conditions and sensitizer absorbance values.

Secondly, CEP is very sensitive to the decay rate of the sensitizer, as any non-radiative decay of excited sensitizers reduces the concentration of sensitizer excitons, greatly reducing the overall CEP yield. However, a simple calculation assuming 1 nm^3^ chromophores at the 1:40 blend ratio used in this work reveals that there are >30 sensitizers within the 5.6 nm Förster radius for Rhod6G self-FRET^29^, indicating that energy transfer among sensitizers before decay is likely and could potentially play a role in counteracting the shortcomings of short-lived sensitizer excitons to boost the efficiency of CEP.

The excitation intensity required for a two-photon, multi-chromophore upconversion process can be roughly estimated using a simple equation: 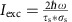, where *τ*_s_ is the lifetime of sensitizer excitons and *σ*_s_ is the cumulative absorption cross-section of all antenna sensitizers that can contribute energy to a common acceptor within an exciton lifetime. This equation suggests two basic pathways towards upconversion at low excitation intensities: long-lived sensitizer states and/or large (effective) absorption cross-sections. CEP provides a platform particularly suited for harnessing long-range energy transport to increase the effective *σ*_s_ and potentially achieve low-intensity upconversion. While CEP itself is a short-range process, the excitations it draws from need not originate from within the domain of CEP action, provided that they can simultaneously encounter an acceptor before decaying. Since nearly every sensitizer has a neighbouring acceptor at optimized CEP blend ratios (especially at the acceptor-heavy blend ratio used in this work), this problem reduces to one of maximizing the probability that two sensitizer excitons encounter each other before they decay. The probability of an encounter between diffusing particles increases with their diffusion rate^30^, so higher exciton mobility yields higher CEP rates. The singlet nature of sensitizer excitons in a CEP system enables highly efficient hopping-type diffusion via both FRET and Dexter energy transfer processes, granting excitons diffusion lengths up to 100 nm^31^ despite their nanosecond-scale lifetimes. In short, CEP happens when two sensitizer excitons encounter each other and the singlet nature of these excitons allows for high mobilities that improve the probability of distant excitons encountering each other. The effective absorption cross-section for CEP therefore includes all of the molecules whose excitons are likely to encounter each other within their lifetimes, up to ∼10^6^ within a 100 nm radius, in the optimal case. This can improve *σ*_s_ far beyond typical single-molecule absorption cross-sections and allow upconversion at lower excitation intensities than would be possible without the benefits of singlet exciton diffusion.

Incorporating the experimental results above into existing analyses of the CEP^15^ and 2PA (ref. 32) processes suggests guidelines for optimizing the selection of sensitizer and acceptor chromophores to maximize the CEP rate (*k*_CEP_), as follows: The acceptor should have a large 2PA cross-section (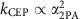) as well as minimal FRET losses to the sensitizers, and it should exhibit minimal self-quenching in the solid state. The sensitizer chromophore should have strong absorbance and also exhibit minimal self-quenching in the solid state. The sensitizer's first excited state should have minimal Stokes shift, strong oscillator strength (*k*_CEP_∝*μ*^4^), and should overlap with 2PA of the acceptor. The morphology of the blended film should have minimal sensitizer-acceptor separation distance (*k*_CEP_*∝r*^−12^).

Further gains can be envisioned through construction of macromolecular antennae assemblies, as recently demonstrated in chromophore-decorated nanoparticle ETU upconversion systems^33^. An ideally designed system would direct excitons toward a common acceptor molecule, analogous to the antenna complex in natural photosynthesis systems that funnels excitations to a reaction centre. This scheme would provide the acceptor with a locally concentrated population of sensitizer excitations, greatly improving the CEP rate over that of an ‘undirected' system at the same excitation intensity.

In conclusion, we have observed singlet-based cooperative energy pooling upconversion in solid-state, air-exposed organic chromophore blends. The proof-of-concept Rhodamine 6G/Stilbene-420 system presented here yielded a 160-fold upconversion improvement over simple two-photon upconversion in Stilb420 and an estimated 0.2% external quantum yield of upconversion under excitation 10^5^ W cm^*−*2^. The estimated internal quantum yield of CEP was 36%, indicating that addressing the numerous energy loss pathways identified in this work may enable dramatic improvements in upconversion. Further optimization of sensitizer-acceptor pairs according to the guidelines listed above can be expected to reveal the full potential of the CEP process to achieve greater and more practical upconversion yields.

## Methods

### CEP film-making procedures

Rhod6G was purchased from Santa Cruz Biotechnology. Stilb420 and Rhodamine 800 were purchased from Exciton (listed as LD800). M540 and RhodB were purchased from Sigma-Aldrich. All materials were used as received. To fabricate thin films Stilbene-420 and Rhodamine 6G were separately mixed into 50 mM solutions in dimethylsulfoxide solvent. These solutions were blended together in a ratio of 40 parts Stilb420 to one part Rhod6G. This blend solution was then coated onto a glass substrate using a Zehntner ZAA 2300 blade applicator with a platen temperature of 105 °C, a blade height of 50 μm and a blade speed of 45 mm s^−1^ to produce films ∼80 nm thick. Glass substrates were cleaned via sonication in acetone and methanol for 5 min each and subsequent UV-ozone treatment for 2 min before film deposition.

### Spectroscopy methods

All absorption data was taken on a Varian Cary 500 spectrometer. Rhodamine 6G emission measurements were taken on a Horiba Scientific Fluorolog spectrofluorimeter using a monochromated Xe lamp as the excitation source. CEP blend film and Stilbene-420 emission spectra were taken on a LaserStrobe spectrometer from Photon Technology International using a GL-3300 nitrogen laser and GL-302 dye laser attachment, also from Photon Technology International. Upconverted emission spectra were measured with the emission filtered by a 500 nm short-pass filter from Thorlabs, model FES0500, to prevent reflected excitation light from interfering with the measured emission signal. Laser power was measured with a 919P-003-10 thermopile sensor from Newport. Time-Resolved Single Photon Counting Data (in Extended Data section) was taken using excitation light generated by a Fianium SC400 supercontinuum fibre laser with wavelength selected by a Fianium AOTF system. Detection was measured via a photomultiplier tube connected to a Becker-Hickl SPC-130 system. All data was collected with signal count rate at <2% of excitation rep rate to ensure proper TCSPC statistics. All spectra were corrected for the spectral responsivities of the systems used for data collection.

### Data availability

The data that support the findings of this study are available from the corresponding author on reasonable request.

## Additional information

**How to cite this article:** Weingarten, D. H. *et al*. Experimental demonstration of photon upconversion via cooperative energy pooling. *Nat. Commun.* **8,** 14808 doi: 10.1038/ncomms14808 (2017).

**Publisher's note:** Springer Nature remains neutral with regard to jurisdictional claims in published maps and institutional affiliations.

## Supplementary Material

Supplementary Information Supplementary Figures, Supplementary Note and Supplementary Reference

## Figures and Tables

**Table 1:** Select CEP parameters and upconversion yields.

	E*	***k***_**CEP**_‡	***k***_**FRET**_‡	***k***_**S**_‡	***k***_**A**_‡	***ɛ***‡	***R***	***d***§	***γ***	**IQY**‖	**EQY**¶
**Solar Flux**	1.7 × 10^3^	1.0 × 10^13^	1.0 × 10^5^	1.0 × 10^8^	1.0 × 10^9^	1.0 × 10^5^	0.50	79	0.50	9.0%	7.0%
**30 × Suns**	5.0 × 10^4^	6.3 × 10^12^	1.0 × 10^5^	1.6 × 10^8^	1.6 × 10^8^	6.3 × 10^4^	0.46	160	0.30	17%	14%
**1,000 × Suns**	1.7 × 10^6^	3.2 × 10^12^	1.0 × 10^5^	2.5 × 10^8^	2.5 × 10^8^	4.0 × 10^4^	0.50	250	0.30	37%	32%
**Best fit to data**#	7.1 × 10^7^–1.2 × 10^9^	9.3 × 10^11^**	1.6 × 10^12^**	4.0 × 10^9^**	2.0 × 10^9^	7.6 × 10^4^	0.025	160	0.30**	3.1–36%#	0.02–0.19%#

The first three rows present internal and external quantum yield (IQY and EQY, respectively) for CEP systems with optimized, yet plausible, system parameters at varying excitation intensities, as calculated by the kinetic model presented in this work. The final row is a best-fit calculation of the quantum yields of the CEP blend film experimentally measured in this work at the range of excitation intensities used in measurement. In the final row the excitation intensity, acceptor decay rate, sensitizer absorption, chromophore blend ratio and film thickness are measured parameters while the CEP rate, FRET rate, sensitizer decay rate and sensitizer self-reabsorption rates are fitting parameters.

^*^Excitation in units of Einsteins nm l^−1^ s^−1^.

^†^Units of s^−1^.

^‡^Units of l mol^−1^ cm^−1^.

^§^Units of nm.

^‖^Internal quantum yield—percentage of absorbed photons that undergo CEP upconversion.

^¶^External quantum yield—percentage of incident photons that are re-emitted at higher energy.

^#^Calculated IQY and EQY values corresponding to the minimum and maximum excitation intensities listed in the first column.

^**^Fitted parameters.
