# Supplementary material for: Experimental demonstration of photon upconversion via cooperative energy pooling
Source: Nat Commun. 2017 Mar 15;8:14808. doi: 10.1038/ncomms14808 (PMC5355946; doi:10.1038/ncomms14808)
Supplement: Supplementary Information — Supplementary Figures, Supplementary Note and Supplementary Reference [file ncomms14808-s1.pdf]

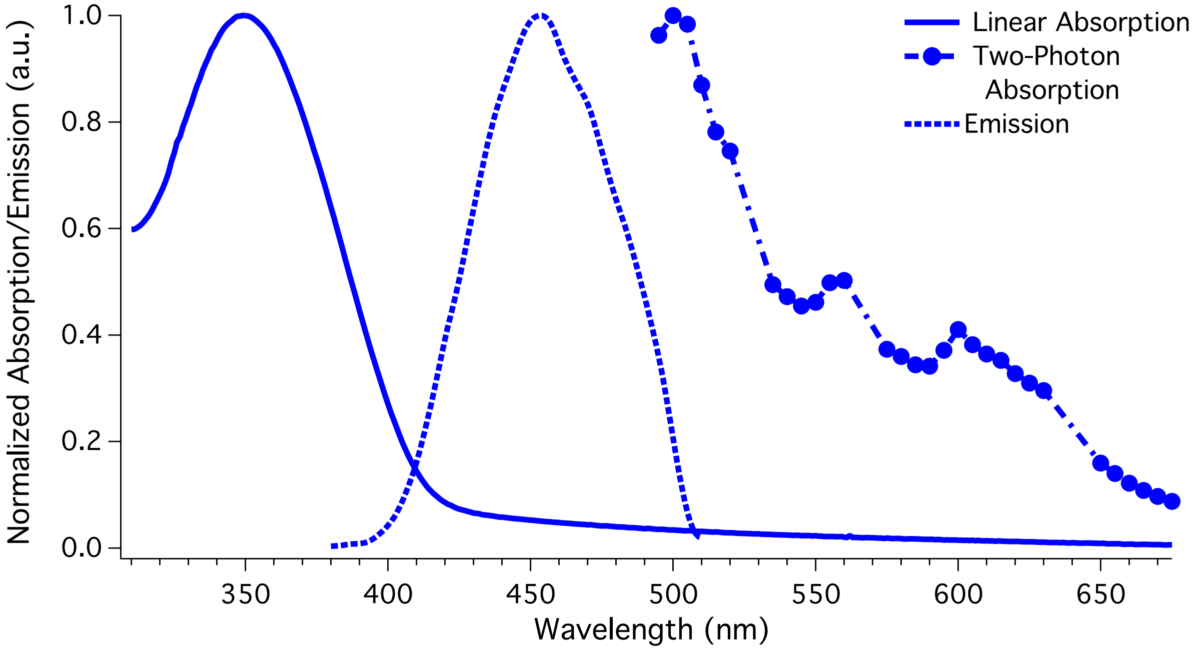

Supplementary Figure 1 | Absorption, emission and two-photon absorption spectra of Stilbene-420. 2PA spectrum was observed in a 100 µmolar solution of Stilb420 in methanol and measured using the LaserStrobe system described in the Methods section.

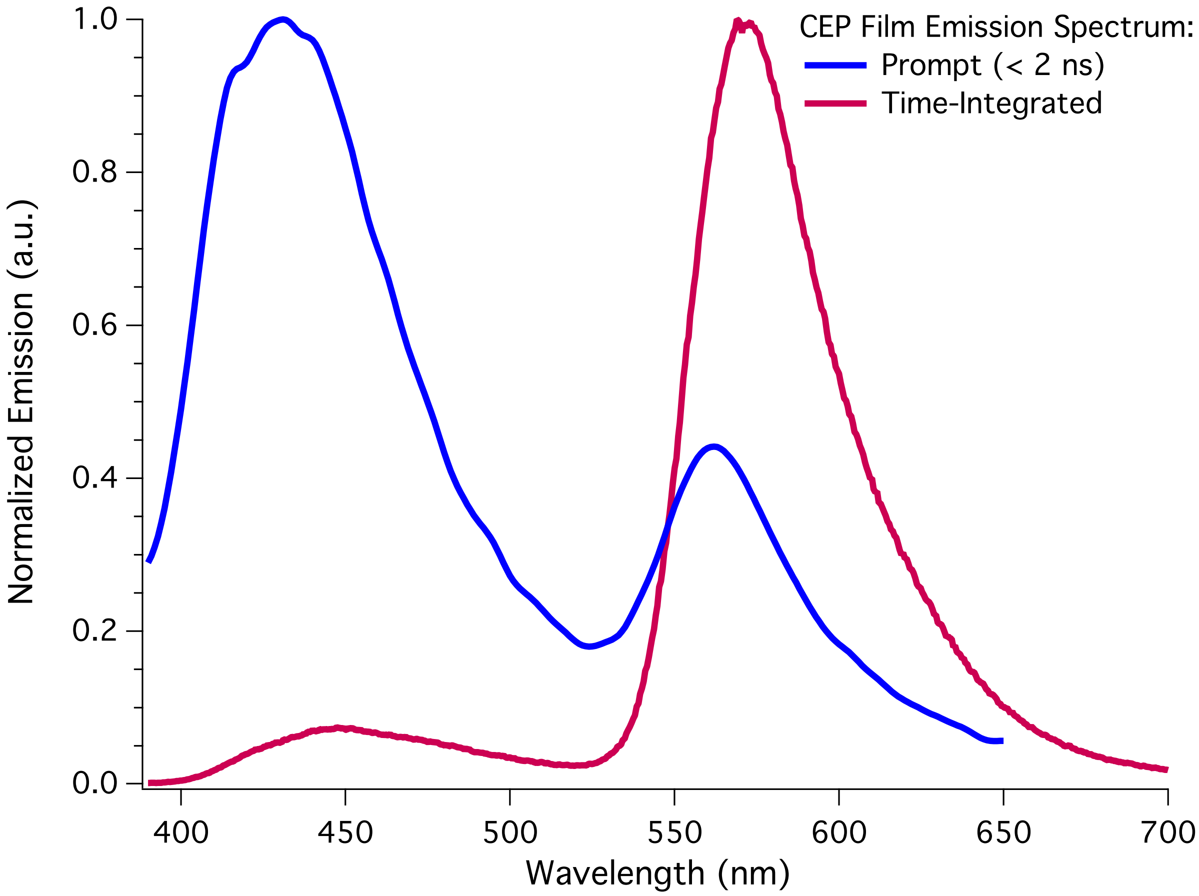

Supplementary Figure 2 | Prompt and time-integrated emission from CEP blend film. At prompt times (< 2 ns) the fluorescence spectrum is dominated by characteristic Stilb420 emission, as expected due to Stilb420 having over five times the absorption of Rhod6G at this excitation wavelength (60,000 $M^{-1}cm^{-1}$ for Stilb420 at 349 nm versus 11,500 $M^{-1}cm^{-1}$ for Rhod6G). Considering that the film is a 1:40 blend of Rhod6G/Stilb420 and that Stilb420 is a better absorber of the excitation light, the strength of Rhod6G emission at prompt times is beyond what one might expect for simple absorption and emission, suggesting energy transfer from Stilb420 to Rhod6G. The time averaged emission spectrum shows that total emission over time is dominated by the Rhod6G emission peak, suggesting that while most absorbed energy is concentrated in Stilb420 at short times it transitions to Rhod6G on a time-scale only slightly longer than the chromophores' decay lifetimes. Taken together, this data suggests that the observed blend film exhibits Förster Resonance Energy Transfer (FRET) from Stilb420 to Rhod6G on a ns-µs timescale.

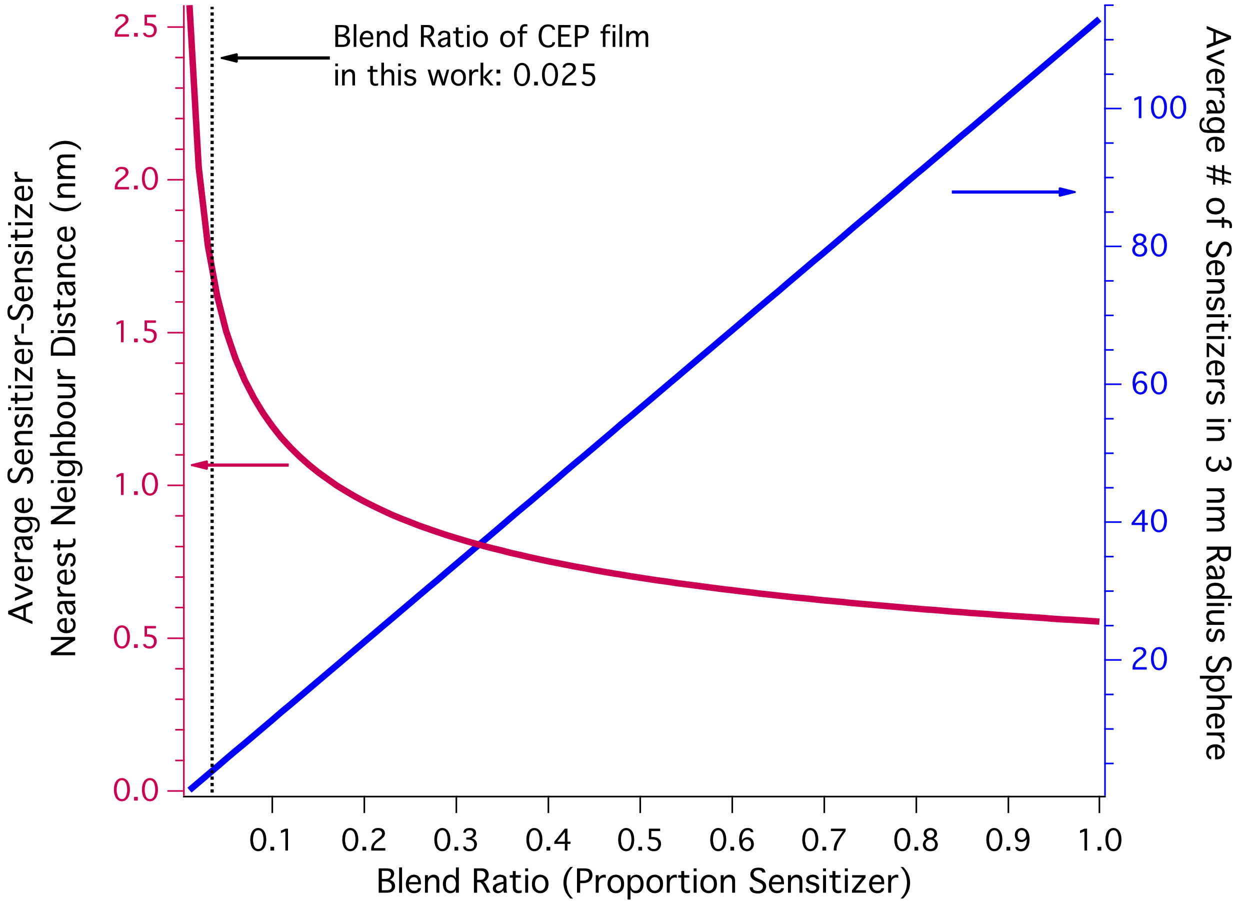

Supplementary Figure 3 | Nearest-neighbour distance as a function of blend ratio. Calculated average sensitizer-sensitizer nearest neighbor distance and average number of sensitizers in a 3 nm radius sphere as a function of chromophore blend ratio. These curves are calculated for a three-dimensional system of point particles whose centres are randomly (Poisson) distributed, following a solution originally by given by Hertz, as reproduced by Torquato[1].

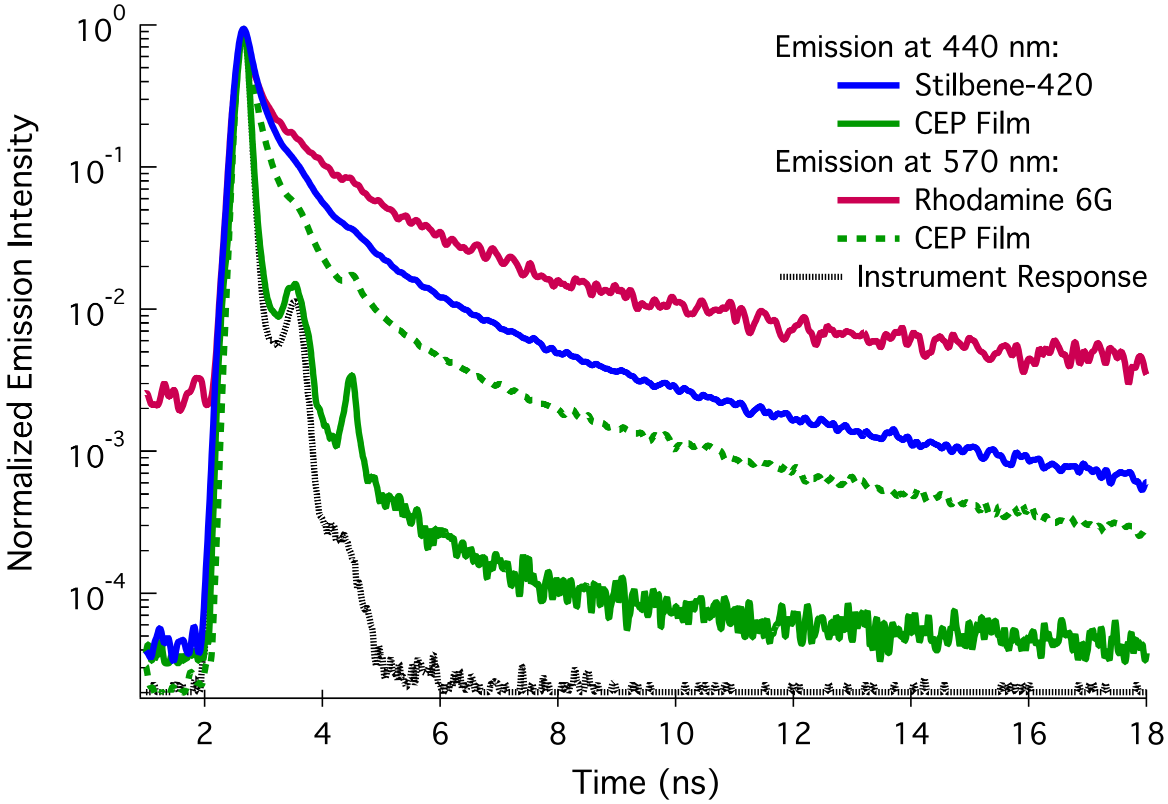

Supplementary Figure 4 | Excited state lifetimes of pristine films and CEP blend film. Time-resolved single-photon counting data on pristine Stilbene-420, Rhodamine6G, and blended CEP film. The upconverted emission from the CEP film at 440 nm was excited with 545 nm light. All other lifetimes were measured using excitation at 400 nm and measuring emission at 440 nm (Stilb420) or 570 nm (Rhod6G and blend film normal emission). Decays were too curved to be accurately fit using mono-exponential lifetimes, but all decays visibly occur on timescales < 1 ns. The reduction in lifetime of both long- and short-wavelength emission from the blend film compared to pristine films indicates the opening of new decay pathways from both Rhod6G and Stilb420 - CEP upconversion and RET, respectively.

Supplementary Note 1 | Determining the Optimal Chromophore Blend Ratio.

The counterintuitive blend ratio of 1:40 (sensitizer:acceptor) was found to be optimal due to strong self-quenching effects in Rhod6G when aggregated. In dilute solution Rhod6G is a strong emitter with peak emission at 552 nm. However, at higher concentrations - both in solution and in film - the emission strength quickly decreases and redshifts. Blend films were fabricated at different ratios. It was found that as the Rhod6G concentration decreased its emission spectrum blue-shifted and approached the dilute solution emission spectrum with a concomitant increase in the CEP yield. Maximum upconverted (CEP) emission was found at a ratio of 1:40, at which point the Rhod6G emission spectrum nearly matched the dilute solution emission spectrum. From this we conclude that Rhod6G self-quenching due to aggregation is the driving factor in determining the optimal blend ratio in this CEP system and that far greater CEP yields may be obtained with a donor chromophore that exhibits less self-quenching losses and allows the film to approach a more optimal blend ratio.

In order to more fully understand the effects of the chromophore blend ratio on the CEP mechanism, we modeled nearest neighbour distances between sensitizers using a Poisson distribution treatment[1]. Included in the extended data is a figure of the average nearest-neighbour distance between sensitizer chromophores as a function of blend ratio, calculated assuming both acceptors and chromophores have spherical volumes of 1 nm^3. This calculation shows that at the 1:40 blend ratio used in this work the average distance between sensitizers is ~1.91 nm. Since all volume not taken up by sensitizers contains acceptor chromophores, the average distance between acceptor and sensitizers is thus closer to one half of the expected sensitizer-sensitizer distance,

or ~0.95 nm. In reality Rhod6G and Stilb420 are closer to 2D discs than to 3D spheres, and the average sensitizer-acceptor distances may be even smaller. Such short separation distances are well within the range of RET, a similar r^{-6}-dependent process, in accord with our observation of CEP at this 1:40 blend ratio. This calculation also indicates that increased blend ratios (i.e. more sensitizers per acceptor) may lead to shorter average separation distances, potentially yielding dramatically improved CEP due to the r^{-6} dependence.

Finally, the Stilb420/PVP control film - used to control for 2PA in pristine Stilb420 - was made at the same weight ratio (rather than molar ratio) as the Rhod6G/Stilb420 film in order to simulate disaggregation of the Stilb420 occurring in the CEP films.

Supplementary References

1. Torquato, S. Nearest-neighbor statistics for packings of hard-spheres and disks. *Phys. Rev. E* **51,** 3170–3182 (1995).
